# Lung Microvascular Niche, Repair, and Engineering

**DOI:** 10.3389/fbioe.2020.00105

**Published:** 2020-02-21

**Authors:** Tomoshi Tsuchiya, Ryoichiro Doi, Tomohiro Obata, Go Hatachi, Takeshi Nagayasu

**Affiliations:** ^1^Department of Surgical Oncology, Graduate School of Biomedical Sciences, Nagasaki University, Nagasaki, Japan; ^2^Division of Nucleic Acid Drug Development, Research Institute for Science and Technology, Tokyo University of Science, Chiba, Japan

**Keywords:** lung microvascular niche, tissue engineering, lung regeneration, decellularization, recellularization

## Abstract

Biomaterials have been used for a long time in the field of medicine. Since the success of “tissue engineering” pioneered by Langer and Vacanti in 1993, tissue engineering studies have advanced from simple tissue generation to whole organ generation with three-dimensional reconstruction. Decellularized scaffolds have been widely used in the field of reconstructive surgery because the tissues used to generate decellularized scaffolds can be easily harvested from animals or humans. When a patient’s own cells can be seeded onto decellularized biomaterials, theoretically this will create immunocompatible organs generated from allo- or xeno-organs. The most important aspect of lung tissue engineering is that the delicate three-dimensional structure of the organ is maintained during the tissue engineering process. Therefore, organ decellularization has special advantages for lung tissue engineering where it is essential to maintain the extremely thin basement membrane in the alveoli. Since 2010, there have been many methodological developments in the decellularization and recellularization of lung scaffolds, which includes improvements in the decellularization protocols and the selection and preparation of seeding cells. However, early transplanted engineered lungs terminated in organ failure in a short period. Immature vasculature reconstruction is considered to be the main cause of engineered organ failure. Immature vasculature causes thrombus formation in the engineered lung. Successful reconstruction of a mature vasculature network would be a major breakthrough in achieving success in lung engineering. In order to regenerate the mature vasculature network, we need to remodel the vascular niche, especially the microvasculature, in the organ scaffold. This review highlights the reconstruction of the vascular niche in a decellularized lung scaffold. Because the vascular niche consists of endothelial cells (ECs), pericytes, extracellular matrix (ECM), and the epithelial–endothelial interface, all of which might affect the vascular tight junction (TJ), we discuss ECM composition and reconstruction, the contribution of ECs and perivascular cells, the air–blood barrier (ABB) function, and the effects of physiological factors during the lung microvasculature repair and engineering process. The goal of the present review is to confirm the possibility of success in lung microvascular engineering in whole organ engineering and explore the future direction of the current methodology.

## Introduction

Biomaterials have been used for a long time in the field of medicine. Since the success of “tissue engineering” pioneered by Langer and Vacanti (1993), tissue engineering studies have advanced from simple tissue generation to whole organ generation with three-dimensional reconstruction. In 2010, investigators in two institutions virtually simultaneously reported the transplantation of a whole organ engineered rat lung (Ott et al., 2010; Petersen et al., 2010). The decellularized rat lung was recellularized by epithelial cells (EpCs) from trachea and endothelial cells (ECs) from pulmonary vessels. The engineered lung was orthotopically transplanted after left lung lobectomy. However, the transplanted engineered lung lost function in a short period. The histology of the failed transplanted lung showed microcapillary thrombus and red blood cell infiltration in the alveolar space (Ott et al., 2010; Petersen et al., 2010). From published studies, it appears that the most crucial problem for whole organ engineering has been the difficulty in the remodeling of a complete vascular network (Stabler et al., 2015). The incompletely remodeled vasculature immediately causes thrombosis in the capillaries and leakage in the alveoli. Further, insufficient blood supply prevents survival of the attached cells in the engineered organs.

In order to reconstruct a complete vascular network, we need to understand the vascular niche composition, which encompasses the ensemble of multi-dimensional relations (i.e., physical, biochemical, and mechanical) between cells and their environment (Soberon and Nakamura, 2009). The lung vascular niche includes not only vascular endothelium and the embedded scaffolds, but also surrounding alveolar epithelium. Importantly, the characteristics and fate of vascular ECs are affected by the attached scaffolds’ architecture and mechanical properties. Scaffolds should sustain embedded EC growth and differentiation, and lead to complete maturation of the vasculature through the delivery of suitable biochemical signals and physiological mechanical stimuli, mediated and translated by the extracellular matrix (ECM) to the ECs. In addition, adjacent or neighboring cells such as alveolar EpCs also affect the property and fate of the ECs. Therefore, the microvascular niche is site specifically established since the surrounding architecture including scaffold and attached cells are extremely different between organs.

As yet, successful reconstruction of lung microvasculature has not been achieved. However, the generation of microvasculature/capillaries is essential for successful lung organ engineering. Therefore, this review focuses on lung microvascular niche reconstruction in whole lung organ engineering. In this review, we cover the following topics: (I) advancements in lung microvascular niche reconstruction. Next, we move to several topics of whole lung organ engineering including (II) the contribution of ECs and perivascular cells, (III) ECM composition and reconstruction, (IV) reconstruction of the air–blood barrier (ABB) function with the role of surfactant protein, (V) the effects of physiological factors during the lung microvascular repair and engineering process, and (VI) evaluating passage functionality of engineered microvasculature. And lastly (VII), in regard of the topics above, we discuss current outstanding questions and prospects, and look at future strategies for lung microvasculature reconstruction. The goal of the present review is to assess the possibility of success in lung microvascular engineering in whole organ engineering and explore future directions of the current methodology.

## Advancements in Lung Microvascular Niche Reconstruction

The knowledge needed for achieving lung microvascular niche reconstruction has been furthered by a variety of studies and research. However, we do not yet know the whole process for achieving successful lung reconstruction. For descriptive purposes, we sort some topics from earlier simplified steps to more complex steps.

### Understanding Lung Microvascular Niche Damage and the Repair Process in Lung Disease

The pulmonary vasculature has specific characteristics of thin walls and easy expandability with high compliance. The pulmonary arterial vascular tree bifurcates from the large diameter pulmonary artery into segmental and subsegmental branches. The branches change to arterioles which terminate in an extensive network of capillaries enrobing the distal alveoli (Stevens, 2011; Stabler et al., 2015). Human large pulmonary arteries consist of elastic laminae, which are separated by smooth muscle and collagen fibers into three distinct layers of tunica intima, tunica media, and tunica adventitia. Medium-sized muscular pulmonary arteries possess a smaller tunica media positioned between internal and external elastic laminae (Horsfield, 1978). Pulmonary arterioles lack the well-defined muscular layer along the proximal–distal axis but still maintain a multi-layer morphology of endothelial and smooth muscle cells. Generally, arterioles function to regulate blood flow volume to the capillaries. Depending on the tissue requirement, the arteriole can expand or collapse and thus control the blood flow. Distal lung capillaries that enrobe the alveoli are comprised of only a single monolayer of flattened ECs supported by pericytes and/or fibroblasts. Capillaries wrap the alveolar internal wall like a sheet and exchange nutrition, ions, hormones, and many other substrates between the blood and interstitial fluid surrounding the cells. Crucially, the gas exchange is performed by distal lung capillaries. Pulmonary venules gather the blood from the capillaries and transport it to the pulmonary veins which flow into the left atrium, providing oxygenated blood to the heart.

According to the site-specific differences of the vasculature network, the vascular endothelium of the lung has some degree of innate functional heterogeneity and vascular niche-specific plasticity (Stevens, 2011). For example, vascular site-specific differences in fluid permeability exist between the larger vasculature and the microvasculature (Parker and Yoshikawa, 2015). Interestingly, lung microvascular ECs (LMVECs) differ from the macrovascular ECs. Microvascular ECs express glycoproteins that are preferentially recognized by Griffonia simplicifolia I lectin which binds to galactose, whereas macrovascular ECs preferentially recognize Helix pomatia lectin which binds to α-*N*-acetylgalactosamine (King et al., 2004). Moreover, LMVECs lack Weibel-Palade bodies, which are ultrastructural hallmarks of other subtypes of ECs, such as pulmonary arteries and arterioles (Ochoa et al., 2010).

**FIGURE 1:**
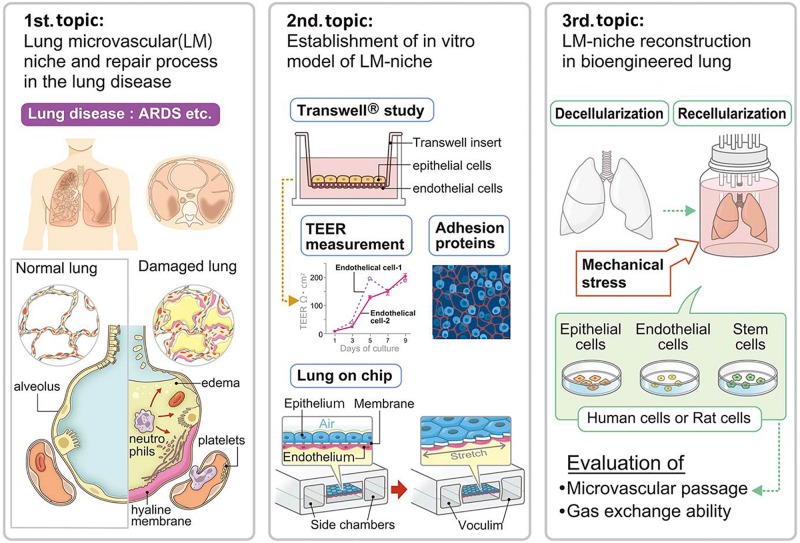
Advancements in lung microvascular niche reconstruction. The lung microvascular niche has been studied in the pathologies of lung disease and animal experimental models. Lung capillary mimics including Transwell^®^ and lung-on-a-chip have been developed by researchers for understanding and simulating the lung microvascular niche. The cell replacement technique for whole lung organ engineering consequently needs to establish a microvascular niche in the natural scaffold. Suitable combination and numbers of cells with ideal mechanical stress will be necessary for whole lung organ engineering, which will be evaluated by microvascular passage, microvascular leakage, and gas exchange ability. ARDS, acute respiratory distress syndrome; TEER, trans-epithelial electrical resistance.

The study of lung damage and repair in diseased lungs has given us many strategies for performing lung microvascular niche engineering because the transplanted engineered lungs are all damaged to some extent and have a very similar pathology to diseased lung (Figure 1) (Doi et al., 2017). A representative lung disease is acute respiratory distress syndrome (ARDS), which is a clinical life-threatening syndrome characterized by an acute onset. Many factors including high oxygen, toxins, and some drugs trigger the disease. Stimulated neutrophils and macrophages in the damaged lung increase the level of cytokines and release mediators including oxygen free radicals, proteases, leukotrienes, and prostaglandins, which damage the endothelium, trigger coagulation, destroy barrier function, and increase vascular permeability. Finally, ARDS results in diffuse alveolar damage (DAD), a pathologically specific condition. Microscopically, there is capillary congestion with thrombi, interstitial and intravascular edema and hemorrhage, necrosis of alveolar EpCs, and collection of neutrophils in capillaries. The alveolar ducts are dilated and alveoli tend to collapse with a secondary impairment of surfactant synthesis. Hyaline membranes, lining the alveolar wall and alveolar ducts, consist of protein-rich edema fluid with remnants of necrotic EpCs. In this situation, both tight junctions (TJs) and adherens junctions (AJs), which play critical roles in maintaining the endothelial barrier function, are disrupted (Luan et al., 2018).

The study of the mechanism of ARDS and the pathology of DAD directly deepens our knowledge of the functioning of the lung niche. Further, the treatment strategy for ARDS, such as respiratory care with non-invasive ventilation or drug therapy using glucocorticoids (Meduri et al., 2016), gives us possible strategies for the reconstruction of the lung niche including the lung microvasculature.

### Establishment of an *in vitro* Model to Study the Alveolar Wall Barrier Function and Lung Vascular Niche

To comprehensively understand the lung vascular niche, researchers have created facsimiles of the lung vascular niche in the laboratory. Studies using *in vitro* systems are important for helping us to understand normal physiology and the effects of added factors including simplified damage. One approach to measure the integrity of the lung vascular niche is the transepithelial/transendothelial electrical resistance (TEER) measurement in a Transwell^®^ assay. This method measures the integrity of TJ dynamics in cell culture models of endothelial and epithelial monolayers (Figure 1) (Neuhaus et al., 2012; Srinivasan et al., 2015; Luan et al., 2018; Yuan et al., 2019). TEER measurements have been used to assess the integrity in such systems as the blood–brain barrier (BBB), gastrointestinal (GI) tract, and pulmonary alveolar septa. In such models, researchers have also studied the immunohistochemical expression of TJ proteins as a measurement of the lung alveolar barrier function. The TJ proteins are composed of transmembrane proteins including occludin, claudin, and ZO-1. In addition, AJ proteins, composed of VE-cadherin and beta-catenin, have also been analyzed. Importantly, a simplified *in vitro* system can clearly show the effects of added factors to key physiological conditions, which gives us hints for successful lung engineering. For example, the Epac-selective cAMP analog 8CPT-2Me-cAMP increased the TEER of iPSC-derived endothelial colony forming cells (ECFCs) (Yuan et al., 2019). Thus, such Epac-agonists might improve epithelial barrier functions during lung organ engineering.

Another approach is the engineering of small lung models that can mimic lung disease conditions and can be used, for instance, to test drugs. Lung-on-a-chip or tiny plastic lungs are biomimetic microsystems which imitate the partial structure of the lungs (Figure 1) (Huh et al., 2010). Lung-on-a-chip was accomplished by micro-fabricating a microfluidic system containing two closely apposed microchannels separated by a thin (10 mm), porous, flexible membrane made of polydimethylsiloxane (PDMS) (Huh et al., 2010). This bioinspired micro-device reconstructs the functional alveolar-capillary interface and reproduces complex integrated organ-level responses to bacteria and inflammatory cytokines introduced into the alveolar space. The models provide low-cost alternatives to animal and clinical studies for drug screening and toxicology applications. Organs on-chips also provide the advantage of enabling the study of cells under physiologically relevant fluid flow conditions that are known to induce mechanotransduction effects on certain cell types (Atencia and Beebe, 2005). The most developed small lung mimetic is a vascularized alveolar model engineered in biocompatible hydrogels. Grigoryan et al. (2019) engineered a multi-vascular network enrobing a 1 cm size air sac with tidal ventilation and showed oxygenation of red blood cells through the vascular network.

These engineered systems represent a simplified lung microvascular niche correlation with EpCs in several physiological situations. Further structural development will be necessary to replace the respiratory function of the lung.

### Lung Microvascular Niche Reconstruction in Bioengineered Lung

In contrast to the idea of engineering *in vitro* lung mimics (second step), whole lung organ engineering trials arose from the idea of modifying from natural biomaterials. The approach is a cell replacement method using recellularization of decellularized organ scaffolds (Tsuchiya et al., 2014b). Currently, a variety of natural scaffolds are available for clinical usage such as arterial grafts, heart valves, urinary tract reconstitution, skin reconstruction, dura mater grafts following intracranial surgery, and orthopedic applications. Such methods have been successful in eliminating intact cells (i.e., decellularize) and degrading nucleic acid remnants to less than 200 base pairs with less than 50 ng of dsDNA per 1 mg dry weight of the ECM scaffold (Gilbert et al., 2006; Crapo et al., 2011; Gilpin and Wagner, 2018). The novel strategy is to reseed the acellular scaffold with a cocktail of appropriate tissue specific cells that can generate functional tissues or organs which have a natural anatomy and which can be orthotopically implanted as is. Accordingly, decellularized whole organ scaffolds preserve the strict organ structure resulting in an acellular and non-antigenic matrix. Thus, theoretically, if the acellular scaffold is reseeded by own cells, immunosuppression following transplantation of the engineered tissue or organ will not be necessary (Gilbert et al., 2006).

The decellularization and recellularization method for providing whole organs for transplantation has significant advantages in whole lung organ engineering. Lung is a dynamic organ with enduring continuous structural change during the act of breathing. Thus, the elasticity and continuity of the air way, which extends from the trachea to the alveoli, must be preserved for efficient respiratory function. In other words, the lung has a specific characteristic in which the lung’s function depends on the organ architecture of the organ itself. Therefore, the engineered lung organ needs the natural branched structure of the native lung in order to provide organ function. In this respect, the cell replacement-based engineering technique is superior to other methodologies. Further, another complicating issue of lung organ engineering is in mimicking the ultrafine structure of the alveoli. Histologically, the human lung consists of 100 billion small balloon-like, extremely thin alveoli sacs (200–500 μm) surrounded by pulmonary capillaries. In healthy human lungs, the average diameter of the alveolar capillary is 8 μm and the alveolar wall thickness is less than 1 μm with a 0.2 μm basement membrane. It is quite difficult to regenerate such a microarchitecture using current technology. Given such a complex organ architecture, lung organ engineering has higher hurdles to overcome than engineering other organs such as liver or pancreas, in which a cell package or organoid can provide partial exocrine functions.

Published studies suggest that the mechanics of the lung microvasculature is maintained following decellularization (Nagao et al., 2013; Tsuchiya et al., 2016) as well as the vessels ability to withstand the majority of the cardiopulmonary output *in vivo* (Song and Ott, 2011). However, functional mature lung microvasculature fabrication has not yet been achieved. Thus, reconstruction of the lung microvascular niche on natural acellular organ scaffolds is the prerequisite for successful whole lung engineering. Given that real microvascular passage has not been achieved in the decellularization and recellularization methods for lung and other organ engineering, complete microvascular niche reconstruction will be the breakthrough methodology needed in order to make functional whole organ engineering possible.

## The Contributions of Endothelial Cells and Perivascular Supporting Cells for Lung Microvascular Niche Reconstruction

Endothelial cells are the main cellular component of the vasculature which separates blood from underling tissues. The role of ECs in the vasculature is more complex than a mere structural role or for simply delivering oxygen and nutrients. ECs modulate the coagulation of blood, regulate the transportation of inflammatory cells, and provide a barrier function (Carmeliet and Jain, 2011; Ghesquière et al., 2014). In addition, the barrier function is strengthened by endothelial supporting cells of pericytes for capillaries and smooth muscle cells for larger vessels. The supporting cells wrap ECs and tighten their adherent and TJs. 3D gel culture studies revealed that the combination of ECs and supporting cells could establish mature vasculature formation *in vitro* (Merfeld-Clauss et al., 2014; Rohringer et al., 2014). Both ECs and the supporting cells produce angiocrine factor, providing inhibitory and stimulatory tissue-specific signals for stem cell renewal (Nolan et al., 2013). Therefore, both ECs and supporting cells are important for reconstruction of the microvascular niche (Carmeliet and Jain, 2011).

Physiological analysis of a decellularized lung scaffold revealed that almost all the perfused flow leaked through the alveolar-capillary membrane and only a residual flow left the vascular circuit through the pulmonary vein (da palma et al., 2015). Results show that suitable reseeding with ECs and supporting cells is necessary for preventing fluid leaking from the alveolar-capillary membrane in vasculature reconstruction and functional organ engineering.

### Endothelialization of Decellularized Microvascular Bed

In the reendothelialization process, ECs attach to the internal lumen of decellularized vascular beds and re-establish the microvascular niche. Following decellularization, a variety of cell populations have been used to reendothelialize the pulmonary vasculature.

Endothelial cells derived from different organs possess significant differences in their genome wide expression levels and also transcriptomes (Nolan et al., 2013). Therefore, theoretically, organ-specific on-site cells might be the most promising cell source for lung microvascular generation. Accordingly, LMVECs have been used for recellularization in rat models (Table 1 and Supplementary Table S1). Niklason’s group have seeded rat LMVEC (RLMVEC) with pulmonary EpCs onto decellularized lung (Petersen et al., 2010; Calle et al., 2016). Human umbilical vein ECs (HUVECs), a non-invasive source of human ECs, also have been used for whole lung engineering (Ott et al., 2010; Gilpin et al., 2014). However, HUVEC had lower levels of proliferation and a higher level of apoptosis compared to LMVECs. In addition, most recellularized HUVECs were located within vessels 11–25 mm in diameter, while LMVECs were most abundant in micro-vessels of 10 μm or less, which may reflect an organ-specific or vessel segment-specific advantage of LMVECs (Scarritt et al., 2018). Accordingly, seeding with three types of cells including pulmonary artery ECs (PAECs) and pulmonary vein ECs (PVECs) with LMVECs into the scaffold influenced the phenotype of the three cells resulting in excellent reseeding morphology, indicating that combination seeding with site-specific cells is ideal (Scarritt et al., 2018).

**TABLE 1:** Summary of organ engineering using decellularized lung scaffolds.

**Spacies of the scaffold**	**Detergents**	**Cell sources for seeding**	**Seeding route**	**Instilled cell number**	**Culture period**
Mouse	8 mM CHAPS	Mouse fibroblast A9 cells	Direct seeding	3500 cells/m^2^–1.0 × 10^6^	1–14 days
	0.1% Triton X-100, 0.1% SDS	Mouse C10 epithelial cells	Airway (Trachea)	1.0 × 10^6^–8.0 × 10^6^	1–28 days
	0.1% Triton X-100, 2% SDC	Mouse fetal lung cells			
		Mouse bone marrow-derived mesenchymal stem cells			
		Mouse embryonic stem cells			
Rat	0.1% SDS	A549 cells	Direct seeding	2000 cells/m^2^–2.0 × 10^6^	1–28 days
	1% SDS	Mouse alveolar type II cells	Airway	0.6 × 10^6^–1.5 × 10^8^	3–21 days
	8 mM CHAPS	Mouse embryonic stem cells	Vascular		
	0.1% Triton X-100, 0.1% SDS	Rat primary pulmonary endothelial cells			
	0.1% Triton X-100, 0.01% SDS	Rat primary epithelial cells			
	0.5% Triton X-100	Rat neonatal lung cells			
	0.5% Triton X-100, 0.01% SDC	Rat lung distal epithelial cells			
	0.5% Triton X-100, 0.05% SDC	Rat lung microvascular endothelial cells			
	0.5% Triton X-100, 0.1% SDC	Rat adipose tissue-derived stem/stromal cells			
	1% Triton X-100, 2% SDC	Human umbilical cord endothelial cells			
	0.15 M NaOH	Human iPSC-derived endothelial cells			
	Potassium laurate	Human iPSC-derived epithelial progenitor cells			
Pig	0.5% SDS	Pig lung distal epithelial cell	Airway	0.5 × 10^6^–1.0 × 10^9^	3–28 days
	1% SDS	Pig bone marrow-derived mesenchymal stem cells	Vascular		
	2% SDS	Human umbilical cord endothelial cells			
	8 mM CHAPS	Human small airway epihtelial cells			
	0.1% Triton X-100, 2% SDC	Human epithelial progenitor cells			
	0.5% Triton X-100, 0.01% SDC				
	0.5% Triton X-100, 0.05% SDC				
	0.5% Triton X-100, 0.1% SDC				
	1%Triton X-100, 0.1% SDS				
Human	0.1%SDS	Rat alveolar type II cells	Direct seeding	2.5 × 10^4^–4.0 × 10^6^	1–12 days
	0.5% SDS	Human primary alveolar type II cells	Airway	1.0 × 10^6^–5.0 × 10^7^	1–30 days
	1% SDS	Human primary pulmonary endothelial cells	Vascular		
	1.8 mM SDS	Human primary pulmonary epithelial cells			
	8 mM CHAPS	Human fetal lung cells			
	3% Tween 20, 4% SDC	Human lung fibroblast			
	0.1% Triton X-100, 2% SDS	Human bronchial epithelial cells			
	0.1% Triton X-100, 2% SDC	Human small airway epithelial cells			
	1% Triton X-100	Human vascular endothelial cells (CBF12 positive cells)			
	3% Triton X-100	Human bone marrow-derived mesenchymal stromal cells			
		Human adipose tissue-derived mesenchymal stromal cells			
		Human iPSC-derived alveolar type II cells			
		Human iPSC-derived endothelial cells			
		Human iPSC-derived epithelial progenitor cells			
Rhesus monkey	0.01% Triton X-100, 0.1% SDS	Rhesus bone marrow-derived mesenchymal stromal cells	Direct seeding	1.0 × 10^5^	8 days
Rhesus macaque	0.1% Triton X-100, 2% SDC	Rhesus adipose tissue-derived mesenchymal stromal cells	Airway (Bronchioles)	1.5 × 10^6^	7 days
		Human embryonic stem cells			

*SDC, sodium deoxycholate. SDS, sodium dodecyl sulfate. CHAPS, 3-[(3-cholamidopropyl)dimethylammonio]-1-propanesulfonate. iPSC, induced pulripotent stem cell.*

Among the immature progenitor and/or stem cells, endothelial progenitor cells (EPCs) isolated from peripheral blood or umbilical cord blood might be a potential source of autologous cells that can be easily harvested (Asahara et al., 1997; Murga et al., 2004). Recent evidence indicates that EPCs contribute to vessel growth both in the embryo and in damaged adult tissues (Carmeliet, 2003). Blood-derived EPCs have already been used in several studies to endothelialize synthetic vascular grafts. In an *in vivo* canine carotid model, implanted EPCs localized on the decellularized vascular bed showed features of a mature EC phenotype 30 days after administration (Rambøl et al., 2018). Liver and pancreas trials also indicate positive results using blood-derived EPCs in organ engineering (Zhou et al., 2016). However, human umbilical cord derived EPCs did not survive in decellularized lung scaffolds compared to HUVECs, suggesting that additional research will be necessary for future application of cells to the scaffold (Ren et al., 2015b).

For generating ECs from stem cells, Cortiella’s group showed that mouse embryonic stem cells (ESCs) differentiated into CD31 positive cells on pulmonary ECM (Cortiella et al., 2010; Nichols et al., 2017). More recently, induced pluripotent stem cells (iPSCs) have been broadly investigated as a promising cell source for ECs (Ren et al., 2015a). iPSC-derived ECs outperformed HUVECs, which are so far considered the gold standard in EC research. Thus, the usage of iPSCs for lung vascular engineering has begun in some institutions. Ott’s group applied human iPSC derived ECs to rat and human lung scaffolds and established perfusable vascular lumens (Ren et al., 2015a). Using a FITC-dextran assay, Niklason’s group showed recellularization by iPSC-derived ECFCs and cAMP enhancement improved endothelial barrier function of engineered rat lung (Yuan et al., 2019). While promising, the clinical use of ECs derived from iPSC is still subject to concerns regarding the tumorigenic potential of pluripotent cells and their limited clinical use (Cossu et al., 2018).

The seeding method itself might affect cell attachment, cell viability, and growth kinetics. For the rat lung scaffold, HUVECs in gravity-flow seeded tissues spread across the vascular matrix, relining the walls of small and large vessels leading to a high recellularization and morphology score. On the other hand, perfusion seeding caused leakage of some cells into the airspace, possibly because of the rupture of vessels during the perfusion process (Scarritt et al., 2018).

### Perivascular Supporting Cells of Pericytes and Vascular SMCs

Although ECs are the main component of the vasculature, ECs supporting cells, pericytes for microvessels, and vascular smooth muscle cells (vSMCs) for larger vessels are also key regulators of angiogenesis and vascular maturation (Carmeliet and Jain, 2011; Neff et al., 2011). For the microvascular including lung capillaries, pericytes share the basal membrane with ECs, and are connected by tight, gap, and adherent junctions. A single pericyte can be connected with several ECs by cell protrusions that wrap around them (Gerhardt and Betsholtz, 2003). Because pericytes can suppress endothelial growth, migration, and microvessel stabilization, pericyte involvement also directly correlates with capillary resistance *in vivo* (Bergers and Song, 2005; Von Tell et al., 2006). Therefore, several studies have focused on the pericyte effect in lung microvascular engineering (Ren et al., 2015a; Doi et al., 2017).

There is a lack of scientific consensus on characterization and standardization in isolation protocols (Ferland-McCollough et al., 2017), but mesenchymal stem/stromal cells (MSCs) have been used as one of the most widely investigated sources from which to derive pericyte and vSMCs. Among the MSCs, adipose derived mesenchymal stem cells (ASCs) might be a suitable cell source because of their ease in harvesting (Pellegata et al., 2018). ASCs can differentiate into a mature smooth muscle phenotype under the right biochemical and biomechanical conditions (Harris et al., 2011; Merfeld-Clauss et al., 2014; Rohringer et al., 2014). The principal factor involved in differentiating ASCs seems to be transforming growth factor-beta 1 (TGF-β1) (Von Tell et al., 2006). Human vSMCs derived from ASCs were seeded onto a small-caliber vascular graft. The resulting vessel wall developed a dense and well-organized structure similar to that of physiological vessels.

For the re-endothelization of the rat decellularized scaffold, Ott’s group introduced the concept of seeding both the endothelial and the perivascular compartments by seeding through both arterial and venous routes (Ren et al., 2015a). They co-seeded either HUVECs and human MSCs (hMSCs), or iPSC-derived ECs and pericytes, and achieved a 75% endothelial coverage with a good barrier function. Re-endothelialized lungs were orthotopically transplanted into rats for 3 days, in an *in vivo* experiment limited to determining the presence of cells and the perfusability of the HUVECs-hMSC seeded grafts (Ren et al., 2015a). Doi et al. (2017) exploited the concept of regenerating the vessel mural compartment by using RLMVECs and rat ASCs which differentiated into pericytes. In the study, cell tracking of the ASCs using quantum dots (QDs655) revealed that QDs655-labeled ASCs expressed pericyte markers of NG2 or PDGFR-β in the ECs co-cultured lung scaffold. They found that pre-seeded ASCs stabilized the EC monolayer in nascent pulmonary vessels, thereby contributing to EC survival in the regenerated lungs. Accordingly, the CD31 positive EC coverage rate was almost 90%. The ASC-mediated stabilization of the ECs clearly reduced vascular permeability and suppressed alveolar hemorrhage in an orthotopic transplant model for up to 3 h. Interestingly, in a preliminary study (Doi et al., 2017), ASCs were shown to migrate to interstitial regions of the alveolar wall while pre-seeded ECs attached to the vascular scaffold and suppressed the migration and recellularization efficacy of ASCs. Although a detailed mechanism has not yet been determined, it seems that particular cells can detect their appropriate location on the scaffold possibly by recognizing distributed adhesion proteins or the composition of the ECM. Moreover, the recellularization strategy itself will affect the arrangement and survival of the attaching cells. For the porcine reendothelialization model, the EC coverage rate reached almost 50% at the lung hilum and 20% at periphery regions without ECs supporting cells (Zhou H. et al., 2018). Pre-seeding of the scaffold with pericyte/vSMCs might improve the attachment and survival of ECs even in larger animal models.

## ECM Composition and Reconstruction

### ECM Composition of the Lung Vasculature

Vasculature ECs are embedded in the lung organ scaffold, which consists of ECM composed of collagen, enzymes, and glycoproteins. The lung ECM not only provides vital physical support or a scaffold for resident cells of the lung and contributes to its mechanical properties including prevention of vessels from collapsing, but the ECM is also essential for the biophysical and biochemical signaling of lung cells (Zhou Y. et al., 2018). Therefore, the ECM is critical for all aspects of vascular biology (Thottappillil and Nair, 2015).

**FIGURE 2:**
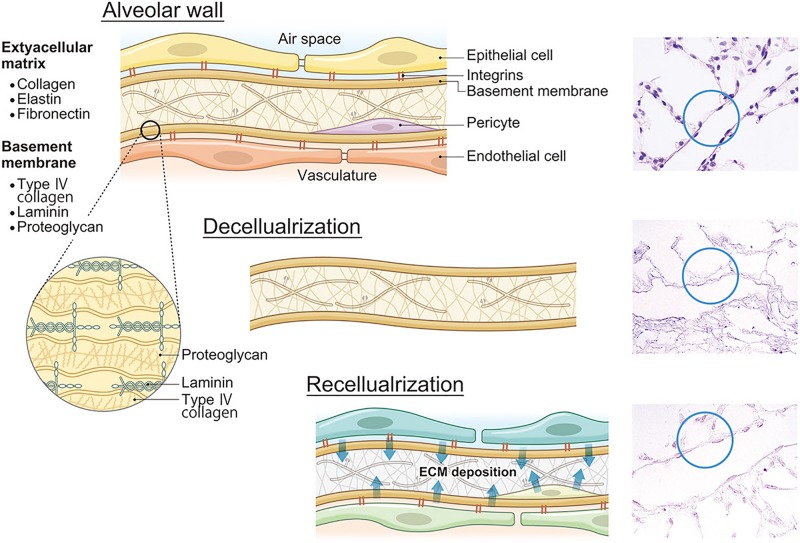
ECM decomposition and reconstruction after recellularization. Lung ECMs consist of collagens, elastin, fibronectin, laminin, proteoglycans, and other constitutive proteins. After lung decellularization, a certain level of ECM damage occurs during decellularization. However, after recellularization, seeded cells secrete own ECM proteins which will change the ECM composition and finally affect lung functions including microvasculature permeability. ECM, extracellular matrix.

In the lung matrix, collagens constitute 15–20% of the dry weight of the lung (Pierce and Hocott, 1960; Burgess and Weckmann, 2012) and many collagen subtypes are found (Thottappillil and Nair, 2015). Fibrillar collagen I and III provide structural integrity (Vandenberg et al., 2000; West, 2013). Collagen IV is the most abundant non-fibrillar network-forming collagen in the lung, which constitutes part of the very thin basement membrane separating capillaries and the alveolar epithelium. This provides stability and tensile strength to the alveoli and the pulmonary capillaries (West and Mathieu-Costello, 2002). Laminins are the primary determinant of basement membrane assembly and other basement membrane components such as collagen IV variants, basement membrane, heparin sulfate proteoglycans, nitrogen compounds, and collagen XVII (Davis and Senger, 2005). Because the basement membrane is fundamental for cell attachment, preservation of natural collagen IV and laminin during the decellularization process might be the most crucial step for not only tissue engineering but also whole lung engineering (Figure 2). There are several other important ECM components. Elastin is secreted mainly by interstitial fibroblasts (West and Mathieu-Costello, 2002) and represents 3–7% of the dry weight of the human lung (McGowan, 1992). Elastin primarily contributes to airway recoil, patency, and parenchymal tethering (Reddel et al., 2012) and also to the elasticity of the pulmonary vascular beds (Yen and Sobin, 2017). Fibronectin is a regulatory multidomain glycoprotein that binds simultaneously to several integrins, which mediate the adhesion of cells, and non-integrin receptors, collagen, and proteoglycans (Roman, 1997). Glycosaminoglycans (GAGs) are unbranched polysaccharide chains composed of repeating disaccharide units. Specific GAGs and proteoglycans play important roles in EC migration and adhesion (Wight et al., 1992).

In the lung microvascular niche, ECM proteins also provide a significant role in a coordinated manner. In quiescent microvessels, pericytes and ECs are embedded in the same basement membrane of collagen IV, laminin, and other components which encase these cells. An interstitial matrix of collagen I and elastin between vascular cells further provides visco-elasticity and strength to the vessel wall (Carmeliet, 2003). In addition, a provisional matrix of fibronectin, fibrin, and other components provides a support scaffold, guiding ECs to their targets.

### Change of Decellularization Protocol Including Detergents

Current lung decellularization procedures have been recognized as causing damage to the ECM thereby altering the capillary function of the engineered lung (Wallis et al., 2012; O’Neill et al., 2013). In order to reduce ECM damage during the decellularization procedure, researchers have improved their protocols including the detergents used for decellularization (Table 1). For the rat lung decellularization, Niklason’s group first used zwitterionic detergents such as 8 mM 3-[(3-cholamidopropyl) dimethylammonio]-1-pro-panesulfonate (CHAPS) solution and then moved to stepwise concentrations of ionic/non-ionic detergent sodium deoxycholate (SDC)/Triton X-100 (Petersen et al., 2010; Calle et al., 2016; Ghaedi et al., 2018). Ott’s group has used the denaturing anionic detergent 0.1% sodium dodecyl sulfate (SDS)/0.1% Triton X-100 (Ott et al., 2010; Gilpin et al., 2014, 2017). Bunnell’s group used 2% SDC/1% Triton X-100 (Scarritt et al., 2013, 2018). Recently, Niklason’s group proposed an advanced protein extraction method using known quantities of proteotypic ^13^C-labeled peptides to quantify matrix proteins in decellularized lung tissues. In the study, SDC/Triton X-100-based decellularization resulted in a near-native retention of the ECM (Calle et al., 2016). Furthermore, the preservation of the ECM may be more important than the removal of residual cells and proteins (Tsuchiya et al., 2014a). Therefore, the SDC/Triton X-100 protocol seems to have the least damaging effect on collagens, elastins, and laminins in both the vascular and airway compartments (Wallis et al., 2012; Stabler et al., 2015; Calle et al., 2016).

Further efforts to improve chemical decellularization showed that NaOH-PBS results in similar structural ECM preservation compared to detergent-based procedures (Sengyoku et al., 2018). Additionally, sulfobetaine-10 (Nagao et al., 2013) and sodium lauryl ester sulfate (Kawasaki et al., 2015) seem to overcome the harmful effects of detergents, such as SDS, on the decellularized vascular ECM. Obata et al. (2019) analyzed the ability of the natural soap potassium laurate, generated from a natural fatty acid, and mainly used for the production of soaps, shampoos, and cosmetics, to act as a detergent for lung bioengineering. The results indicated that potassium laurate has the advantages of safety and efficacy including ECM preservation, survival of the seeded cells, and lower immunoreactivity for organ bioengineering (Obata et al., 2019).

### Change of Decellularization Procedure

In the native human lung, the pulmonary capillary pressure is very low, varying from 6 to 8 mmHg. Thus, high pressure perfusion decellularization in the pulmonary vasculature induces significantly increased stress conditions, causing perforation of the fragile capillaries (West, 2013). Therefore, the detergent application methods have been refined. Monitoring/controlling perfusion via the pulmonary artery resulted in improved maintenance of the pulmonary vascular ECM (da palma et al., 2015). The decellularization route was also changed. In most organs (e.g., heart, kidney, liver), the only available route is through the circulatory bed of the organ. The lung, however, has an additional possible route for decellularization since the detergents can also be infused through the trachea. In fact, both routes (trachea and pulmonary artery) alone or combined have been used to effectively decellularize lungs (Price et al., 2010; Maghsoudlou et al., 2013; Tsuchiya et al., 2016; Wang et al., 2016). By using an intermittent intra-tracheal flow of detergent for decellularization, the same combination of SDC/Triton X-100 yielded an acellular scaffold in a shorter time with an improved preservation of pulmonary micro-architecture (Maghsoudlou et al., 2013). In addition, administrating detergent via the trachea using a bioreactor, which is a ventilation-based decellularization system with negative pressure and positive end-expiratory pressure, results in a uniform decellularized scaffold. In this system, the negative pressure in the bioreactor opens the peripheral airway and spreads the detergents to the rest of the lung (Tsuchiya et al., 2016). Improvements in the decellularization protocols including the use of better decellularization solutions and procedures now appear to be successful in maintaining vascular scaffold preservation for vascular beds.

### Matrix Reconstruction

Approximately 2–10% of the collagen in an adult human lung is renewed every day (Townsley, 2012; Calle et al., 2014). That indicates that a certain level of ECM damage might be repaired by the re-seeded cells. It has been shown that implanted cells, such as ECs, pericytes, MSCs, etc., which were used to engineer the pulmonary vasculature, repaired/rebuilt the adjacent ECM (Davis and Senger, 2005; Zhou Y. et al., 2018). One study showed that an increase in collagen synthesis and elastin synthesis began within 4 h when mechanical tension was applied to explants of rings of a rat pulmonary artery (Tozzi et al., 1989). This occurs in human disease. Mitral stenosis causes pulmonary hypertension, which induces thickening of the basement membrane and ECM of the pulmonary capillaries (West, 2013). Accordingly, following implantation of tissue-engineered vascular grafts re-populated with murine MSCs in an inferior vena cava interposition model, the seeded cells produced collagen I, III, and IV, elastin, fibrillin, and GAGs over a 4-week period. In our preliminary rat study, GAG levels partially recovered following reseeding with RLMVECs and adipogenic stem/stromal cells (ASCs) after 7 days of recellularization. Further, the GAG levels generated by ASCs were clearly higher than those produced by RLMVEC cells alone. This indicates that re-seeded cells can regenerate the ECM; the characteristics and amount of replaced ECM might depend on the species and the type of cells used for re-cellularization (Hashimoto et al., 2019).

It is also well known that ECM degradation easily occurs during the tissue or organ engineering process. Matrix turnover involves both extracellular proteolysis and cell-mediated uptake of cleaved matrix fragments (McKleroy et al., 2013). Various proteolytic enzymes, such as the matrix metalloproteinases (MMPs), and their endogenous inhibitors, tissue inhibitors of metalloproteinases (TIMPs), are involved in re-modeling the ECM during development and in ECM homeostasis. The fetal lung is characterized by a greater proteolytic profile (higher MMP-2 and less TIMP-3 expression), while the adult lung is more anti-proteolytic (less MMP-2 and greater TIMP-3) (Zhou Y. et al., 2018). Our previous study demonstrated that an RLMVEC pulse ASC-recellularized engineered lung has 7016 times higher MMP-2 mRNA expression than RLMVEC alone re-cellularized engineered lung, suggesting that ASCs have strong tissue remodeling efficacy (Doi et al., 2017). Consistently, Ott’s group applies a two-phase culture method for rat lung re-cellularization (Ren et al., 2015a). They use angiogenic medium containing phobol-12-myristate-13-acetate at the beginning of the culture and subsequently use stabilization medium for the prevention of vascular re-modeling. Given the evidence of matrix reconstruction, it is apparent that careful selection of seeding cells and their incubation time will be necessary for whole lung engineering because unsuitable matrix reconstruction will cause microvasculature architecture destruction and result in capillary obstruction.

## Reconstruction of the Air–Blood Barrier (ABB) Function

**FIGURE 3:**
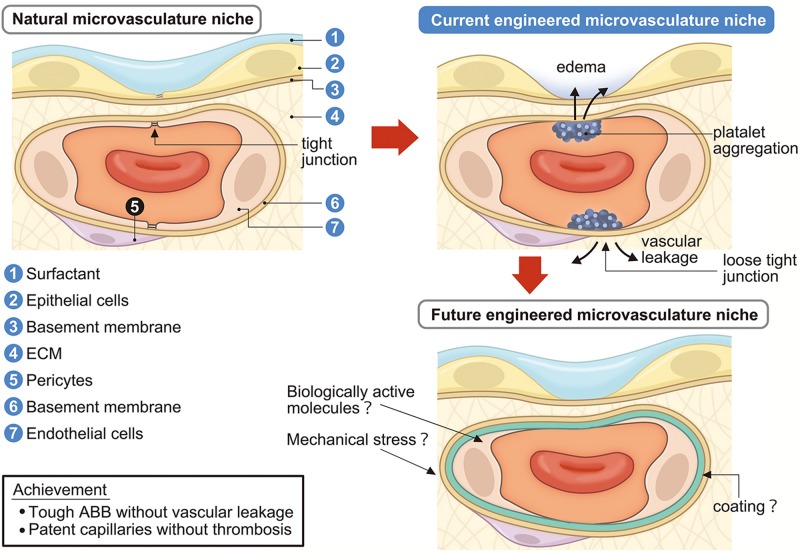
Engineering of lung microvascular niche in the decellularized scaffold. Current engineered microvascular niches consist of incomplete epithelialization and endothelialization which causes vascular leakage, edema, platelet aggregation, and coagulation. Future engineered microvascular niches will achieve a strong ABB without vascular leakage and passable capillaries without coagulation by administrating surfactant, biologically active molecules, mechanical stresses, and/or suitable coating. ECM, extracellular matrix; ABB, alveolar-blood barrier.

Alveolar septa, which are 1 μm or thinner, consist of capillary endothelium and alveolar epithelium with their underlying basement membranes. In addition, the interstitial space of ECM and surfactant protein covering the alveolar inner lumen are also important components of the alveolar septa (Figure 3). The alveolar septa not only perform the essential function of the lung which is to exchange oxygen from the inhaled air and carbon dioxide from venous blood, but the alveolar septa also function as a strong ABB. The ABB consists of the lung endothelial barrier and the alveolar epithelial barrier (AEB). The ABB shields the lung from harmful external elements, like inhaled toxins, particles, or microorganisms. The ABB also strictly regulates permeability and material transfer between blood and tissue. Because the ABB maintains vascular permeability (Bhattacharya and Matthay, 2013), dysfunction or hypo-constitution of the ABB easily causes plasma component leakage into the stroma or alveolar space, which induces lung edema resulting in respiratory failure. For successful whole lung engineering, the reconstruction of an intact and functioning ABB will be crucial for preserving respiratory function.

### The Lung Endothelial Barrier

The vascular endothelium is the first barrier in the ABB, which is called the “lung endothelial barrier” (Bhattacharya and Matthay, 2013). In the normal state, vascular permeability is kept low in tissues. When inflammation occurs, vascular permeability increases as a biological defense mechanism and leakage of plasma components and immune cell migration are induced (Figure 3). Material transfer across the vascular wall occurs via the transcellular pathway and paracellular pathway (Komarova and Malik, 2013). The transcellular pathway is regulated by Caveolae-dependent endocytosis and the paracellular pathway is regulated by cell–cell adhesion of ECs, which consists of AJs and TJs (Dejana, 2004).

In quiescent vessels, vascular endothelial cadherin (VE-cadherin) in AJs and claudins, as well as occludin and JAM-1 in TJs, provide mechanical strength and tightness and establish a permeability barrier (Dejana, 2004). These molecules serve not only as gate keepers, but they also transmit essential signals for endothelial survival, cell growth inhibition, and other functions (Carmeliet et al., 1999). Among the junction proteins, the AJ constitutive main adhesion factor of VE-cadherin plays an important role (Dejana, 2004). Vascular endothelial growth factor (VEGF) increases vascular permeability by destroying the adhesion of VE-cadherin. Weakening or loss of cadherin interactions at AJs accounts for the essential molecular defects underlying endothelial barrier failure (Bhattacharya and Matthay, 2013). On the other hand, Angiopoietin-1 (Ang1), lipid mediator sphingosine-1-phosphate (S1P), the intracellular second messenger of cyclic AMP, and the cAMP activating substrates are known to reduce vascular permeability by strengthening the adhesion of VE-cadherin (Fukuhara et al., 2004). ECs also express neural cadherin (N-cadherin) at the basal membrane side, which mediates binding to pericytes or other mesenchymal cells.

The successful re-establishment of barrier function with adequate cell–cell junctions in recellularized lung microvascular has not yet been achieved. Niklason’s group has focused on the endothelial barrier function using HUVECs and iPSC-derived ECFCs along with endothelial barrier enhancing chemicals (Yuan et al., 2019). Among the added chemicals, Epac-selective cAMP administration induced VE-cadherin and zona occludens-1 (ZO-1) expression at the cell membrane and increased TEER values to the level of HUVECs during *in vitro* culture. Further, they confirmed the barrier function of engineered rat lung microvasculature using a FITC-dextran assay and found improved vascular function in the engineered rat lung (Yuan et al., 2019). The results indicate that the appropriate chemicals can induce TJ formation and enhance ABB function in an engineered lung. However, in this study, the TEER level achieved in the *in vitro* culture was at most 10 Ωcm^2^, which is not sufficient for a fully functional lung (Luan et al., 2018).

In an engineered rat lung transplantation model (Doi et al., 2017), co-seeding of RLMVEC and ASCs prevented alveolar hemorrhage after transplantation, which indicates that the endothelial barrier can be re-established in the engineered lung using ASCs. Gene expression profiling in the engineered lung revealed significantly changed gene expression of components of the angiogenesis-related ANG/TIE signaling system when ASCs are added. The ANG-1 gene, which is expressed by mural cells and stimulates mural coverage and basement membrane deposition with promoting vessel tightness (Carmeliet and Jain, 2011), was strongly upregulated. In contrast, Tie1 and Tie2/Tek, which are essential for EC proliferation, migration, and survival during angiogenesis, were downregulated. This implies that ASCs potentially promote the maturation and quiescence of reconstructed lung microvessels rather than the induction of angiogenesis.

Bunnell’s group confirmed the barrier function by bovine albumin conjugated Evans blue dye extravasation and found that leakage was similar between native rat lung and engineered rat lung with sequential seeding of MVECs, PA-ECs, and then PV-ECs (Scarritt et al., 2018). Combination seeding of these cells led to positive VE-cadherin staining, indicating the formation of TJs between ECs. However, leakage was observed in the distal portions of the re-endothelialized tissue suggesting that recellularization of the alveoli is necessary to completely restore barrier function of the capillary–alveolar network.

### The Alveolar Epithelial Barrier

The alveolar epithelium also has a barrier function (Bhattacharya and Matthay, 2013). Lung epithelium consists of 40 or more different resident cell types (Franks et al., 2008). But the normal AEB is composed of alveolar epithelial type I (AT1) cells and alveolar epithelial type II (AT2) cells (Crandall et al., 2001). The main cause of fluid leakage is the space between the cells and disruption of the TJ between ATI and AT II cells, which directly influence permeability of lung vasculature.

A trans-epithelial electrical resistance (TEER) measurement study revealed that TEER values of primary cultures of rat type II pulmonary EpCs have been cited as high as 2000 Ωcm^2^, which indicates enough AEB function. On the other hand, in a pulmonary EpC model for drug metabolism, the TEER measurement of adenocarcinoma originated A549 cells was 300–700 Ωcm^2^ (Foster et al., 1998). An immortalized human ATI cell line (TT1) does not appear to develop tight intercellular junctions (Kuehn et al., 2016). Human alveolar epithelial lentivirus immortalized (hAELVi) cells maintain the capacity to form tight intercellular junctions, with high TEER values (>1000 Ωcm^2^) (Kuehn et al., 2016). These results suggest that the AEB function deeply correlates with cell source.

From the lung *ex vivo* vascular permeability studies, ensuring the function of the vessel barrier by alveolar epithelium is extremely important for controlling fluid leakage from capillaries to the alveolar space, because the epithelial barrier takes over the endothelial barrier function (Parker and Yoshikawa, 2015; Ren et al., 2015a; Scarritt et al., 2018; Yuan et al., 2019). Theoretically, the best AEB function will be re-established in decellularized organ scaffolds by administrating natural cell types and in sufficient cell numbers. In order to achieve this goal, Niklason’s and Ott’s groups harvested and used dissociated whole lung cells from the donor lung in a rat model (Table 1) (Ott et al., 2010; Petersen et al., 2010). Cortiella’s group cultured autologous cells harvested from a resected own left lung and seeded an acellular pig lung for pig bioengineered lung transplantation (Nichols et al., 2018). However, for humans, it is impossible to harvest whole own lung cells from biopsies. Other candidates for epithelialization of alveoli are immature stem/progenitor cells including ECs, iPS cells, KRT5 + TP63 + basal epithelial stem cells, focusing on the differentiation potential for lung EpCs (Gilpin et al., 2016). Niklason’s group developed iPS derived ATII cells and showed that exposing induced ATII cells to the Wnt/β-catenin inhibitor (IWR-1) changed the iPSC-ATII-like phenotype to a predominantly ATI-like phenotype. Acellular rat or human lung scaffolds were seeded with the cells and the cells displayed markers of differentiated pulmonary epithelium. The minimal cell type combination and the best administration and timing of re-seeding cells should be explored along with the goal of obtaining a mature AEB with tough alveolar junctions and TJs.

### Alveolar Surfactant Protein

Alveolar surfactant protein covers the internal lumen of the alveolar space and also has a significant effect on maintaining normal lung fluid balance within the ABB. Surfactant deficiency increases alveolar surface tension and causes negative pressure surrounding pulmonary capillaries that could promote fluid filtration leading to pulmonary edema (Raj, 1987). In infant respiratory distress syndrome, a deficiency of pulmonary surfactant results in an increased capillary transmural pressure and causes capillary bleeding. In this condition, the capillary pressure itself is not raised but the pressure in the interstitium around the capillaries is reduced because of the rise in surface tension of the alveolar lining layer (Roman, 1997). Despite the importance of the lung fluid balance within the ABB, there is no information about the impact of alveolar surfactant protein on the ABB in bioengineered lung transplantation.

## The Effects of Physiological Factors During the Lung Microvascular Repair and Engineering Process

It has been shown that physiological factors affect the lung microvascular repair and engineering process. Respiratory movement of the diaphragm and auxiliary breathing muscles continuously repeat inflation and deflation of the lung. Thus, the lung is considered a dynamic organ. Cyclical strain during the breathing motion and the pulsatile nature of blood flow exposes blood vessels to mechanical stress (Gray and Stroka, 2017). Two main forces of mechanical stress on the lung vasculature are the frictional force from blood flow (i.e., shear stress) that is parallel to the vessel wall, and the transmural force from blood pressure (i.e., cyclic stretch) that stretches the vessel wall (Lindsey et al., 2014; Baeyens et al., 2016). The endothelium converts these mechanical stresses to biochemical signals that regulate gene expression and cell behavior, including proliferation, migration, remodeling, permeability, and apoptosis. There is evidence that mechanical stress on the lung relates to the lung microvasculature maintenance, repair, and engineering process. In addition, oxygen tension is known to affect the differentiation of the cells (Garreta et al., 2014), which might be another important factor for lung repair and the engineering process.

### Shear Stress and Cyclic Stretch on the Lung Vasculature

Under physiological range shear stress, endothelia elongate and align in the direction of flow, and redistribute junctional proteins (Gray and Stroka, 2017). The ability of cells to sense the direction and strength of shear stress has been suggested to occur via specialized mechanisms and pathways, involving membrane receptors such as integrins, VEGF-2, receptor tyrosine kinases (RTKs), VE-cadherin, PECAM-1, and ion channels (Chen et al., 2002; Orr et al., 2006; Baeyens et al., 2016; Gray and Stroka, 2017). Physiological range shear stress on ECs (10–20 dynes/cm^2^) (Sakariassen et al., 2015) also stabilizes blood vessels (Nigro et al., 2010; Zhou et al., 2014) and has been associated with barrier enhancement, suggesting that shear stress is important in normal barrier maintenance (Birukov et al., 2002). In addition, shear stress reduces EC turnover through inhibition of cell proliferation and suppression of apoptosis via activation of p53 and the PI3K-Akt survival pathway, and nitric oxide production.

Cyclic stretch is another important mechanical force. Cyclic stretch is produced by pulsatile distention of the arterial wall, or by tidal breathing. Blood pressure is the major determinant of vessel cyclic stretch. Similar to shear stress, cyclic stretch induces reorientation of the endothelium, but transverse to the direction of strain (Birukova et al., 2006; Birukov et al., 2015). Cultured ECs stretched at 5% cyclic stretch for 2 h activate Ras-related C3 botulinum toxin substrate 1 (Rac) signaling and induces redistribution of actin and cortactin (an actin-binding protein) toward the cell periphery (Birukova et al., 2004; Birukov et al., 2019). In addition, cyclic stretch in 12 h of normothermic *ex vivo* lung perfusion (EVLP) also significantly influences pulmonary vascular resistance and vascular integrity (Cypel et al., 2008).

### Application of Mechanical Stress for Lung Microvascular Engineering

Usage of mechanical stress in lung vascular engineering is of particular interest since cells in the lung are subjected to breathing-induced cyclic stretch in combination with shear stress from blood flow within the lung alveoli (Gray and Stroka, 2017). The effects of mechanical stress on cell behavior have been investigated by application of biomimetic on-chip and microfluidic platforms (Dongeun et al., 2012). In opposition to traditional *in vitro* cell culture, these microfluidic devices (MFDs) provide the opportunity to integrate multiple mechanical stresses (e.g., shear stress, substrate stiffness) with *in situ* quantification capabilities. They can be used to recapitulate the *in vivo* mechanical setting and systematically vary microenvironmental conditions, using minimal resources, for improved mechanobiological studies of the endothelium (Gray and Stroka, 2017).

The effects of mechanical stress on EC behavior have been investigated by integrating stretch capabilities into MFDs (Simmons et al., 2013; Mahler et al., 2014; Jufri et al., 2015; Mina et al., 2017; Russo et al., 2018). For instance, Zheng et al. (2012) created a microfluidic flow-stretch chip to study shear stress (26 dyn/cm^2^), cyclic stretch (5%, 1 Hz), and the combined effects on ECs HUVEC cultured on the chip showed enhanced cell adhesion and spreading on an artificial surface in all conditions relative to static controls. Dongeun et al. (2012) created a biomimetic microsystem “lung on a chip” that reconstitutes the functional alveolar-capillary interface of the human lung, incorporating cyclic stretching and fluid shear stress. This system was used to test the effect of cyclic mechanical stress on the transport of silica nanoparticles. This study also revealed that mechanical stress enhances epithelial and endothelial uptake of nanoparticles and stimulates their transport into the underlying microvascular channel (Marturano-Kruik et al., 2018). Stucki et al. (2015) showed that the functionality of the alveolar barrier could be restored by co-culturing epithelial and ECs that form tight monolayers on each side of a thin, porous and stretchable membrane in the lung-on-a-chip system. They also showed that cyclic stretch significantly affects the permeability properties of EpC layers in that system (Stucki et al., 2015). Grigoryan et al. (2019) created a 1 cm size vascularized alveolar model that achieved oxygenation of red blood cells in the vascular network with tidal ventilation in the air sac. This model could provide a means of determining the effect of mechanical stress on the vascular network.

According to studies using MFDs or organs on a chip, the two types of mechanical stimuli of pulsatile fluid pumping shear stress and breathing cyclic stretch will be analyzed to determine the seeded cells’ response and establish their functions in the bioengineered lung model, since stress fiber alignment was weakly induced by shear stress, but strongly organized parallel to flow with the addition of cyclic stretch, suggesting a synergistic effect (Gray and Stroka, 2017). Although a variety of bioreactors have been developed to subject the cells to combinations of mechanical stress, none have succeeded in determining the effects of mechanical stress on the seeded cells or their established function such as that of the ABB during *ex vivo* whole lung engineering (Panoskaltsis-Mortari, 2015).

### Oxygen Tension for Lung Cell Differentiation and Lung Microvascular Engineering

Typically, lung regeneration studies have been carried out at the normal atmospheric oxygen level of 20% in air culture chambers. However, O_2_ levels in the *in vivo* cell microenvironment, depending on the tissue, are much lower. For example, O_2_ is about 13% in the arterial endothelium and 2–5% in cells of healthy tissues. Campillo et al. (2016) fabricated a novel chip approach, in which cells cultured on top of thin PDMS membranes can be subjected to fast changes in oxygen concentration. Intermittent hypoxia applied to the chip inhibits the degradation of hypoxia-inducible factor 1α (HIF-1α) and promotes HIF-1 activation in rat bone marrow derived MSCs (Campillo et al., 2016). The same system might affect the remodeling of the lung vasculature via the Akt/mTOR/HIF-1α signaling pathway (Liu et al., 2019). Interestingly, using an oxygen concentration of 5% compared to 20% oxygen, significantly upregulates Nkx2.1 and Foxa2, which are endodermal and early lung EpC markers in ESCs and iPSCs. Further, hypoxic priming of mouse ESCs highly increased HIF-1 mediated VEGF, and efficiently differentiated mouse ESCs to ECs without the need of adding exogenous growth factors (Lee et al., 2012). Accordingly, low oxygen tension might exert a significant positive effect on lung cell differentiation and lung microvascular niche reconstruction especially for ESCs or iPSCs derived progenitor cell repopulation on acellular lung scaffolds (Garreta et al., 2014).

## Evaluating Passage Functionality of Engineered Microvasculature

The success of lung microvascular niche engineering will be evaluated by measuring the passage and leakage of suitable sizes of particles through re-endothelialized lung alveolar capillaries. In a rat model, Ott’s group has shown that 0.2 μm microsphere particles passed through an iPSCs derived peripheral regenerated vascular network 3 days after the engineered lung transplantation (Ren et al., 2015a). Sengyoku et al. (2018) has shown that 0.25 μm artificial red blood cells successfully passed thorough HUVEC re-endothelialized rat lung capillaries in the bioreactor. Therefore, it has been demonstrated that at least 0.2–0.25 μm microsphere particles can pass through re-endothelialized lung with rat size microcapillaries.

However, detailed analysis of dual seeding of cells into the arteries and veins showed that HUVECs did not pass through the rat decellularized rat lung capillaries presumably because of size restriction (Carmeliet et al., 1999). For the evaluation of vascular leakage, Tsuchiya et al. (2016) used human red blood cells to analyze the integrity of decellularized rat microcapillaries. Bunnell’s group used BSA-dye for analysis of extravasation from engineered vasculature and evaluated the most mature vasculature after the rat lung recellularization (Scarritt et al., 2018). In evaluating a human size engineered vascular bed, Ott’s group recently reported recellularization of human cells on a pig lung scaffold (Zhou H. et al., 2018). By repopulating a pig acellular lung with HUVECs, they generated a pulmonary vasculature with mature endothelial lining and sufficient anti-thrombotic function to enable preservation of physiological blood flow and maintained the recipient’s pulmonary circulation and respiratory function during a 1-h observation. However, there is no direct evidence regarding passage and leakage using micro-particles or dye analysis possibly because of technical difficulties.

## Future Strategies for Lung Microvasculature Reconstruction: Outstanding Questions and Prospects

One of the goals of lung organ engineering is to generate functional capillaries with a healthy microvascular niche, which will preserve blood passage through capillaries and also reconstitute the natural barrier properties of both the vasculature and the airway compartments. To achieve the goal of lung organ engineering, there are two different strategies available at present. One is to simulate infant lung development in a 3D-culture system in a bioreactor over a relatively long period. This strategy would mimic embryonic development using immature cells to engineer a mature organ. During the process, many trophic or growth factors will be necessary for long-term *ex vivo* culture in order to induce organ maturation. The drawback of this strategy is that a variety of cytokines are produced by co-cultured cells, which cannot be identified or precisely controlled. Further, a long-term culture will rearrange the ECM by cell-secreting proteins such as matrix metalloproteases (MMPs) and that might harm lung conformation including that of the vascular niche. This strategy also needs long-term infection control. Therefore, the approach for long-term complete monitoring of the culture with the perfect simulation of a stepwise embryonic environment in the bioreactor is underway (Engler et al., 2018).

The other potential strategy is to engineer lungs in a short period (i.e., several weeks to months) using mature cells. This strategy arose from a more conventional engineering approach which aims to fabricate lung from several biomaterial components. Immature cells or dividing cells induce ECM matrix reconstruction and microstructure alteration. Thus, a quiescent vasculature condition should be maintained after the reseeding of the cells. Therefore, short-term culture of mature cells has the advantage that it avoids ECM destruction and preserves a more natural alveolar architecture (Carmeliet and Jain, 2011). However, until now, reseeded ECs have only been able to achieve at most 75–90% coverage of the ECM in a rat model (Ren et al., 2015a; Sengyoku et al., 2018), 20–50% in a porcine model (Zhou H. et al., 2018), and 75% in a human model (Ren et al., 2015a). Unfortunately, blood coagulation and thrombus will occur in the remaining uncovered vascular bed. In addition, we do not know if natural TJ can be established from iPS or ESCs derived ECs. These limitations have led us to conclude that the establishment of complete re-endothelialized vasculature is quite difficult to achieve by recellularization only.

The answer to successful reseeding might be to coat the vascular ECM prior to or post cell seeding. Jensen et al. (2012) attempted to compensate for the loss of or damage to ECM/BM proteins by precoating the decellularized scaffolds with defined adhesion promoting proteins, such as collagen I or Matrigel (Price et al., 2010; Lecht et al., 2014). Wagner’s group used calcium alginate as an artificial pleura to prevent different cell types including EpCs, lung fibroblasts, and pulmonary vascular ECs to leak out of decellularized tissue during seeding due to the loss of pleura upon dissection (Wagner et al., 2014). Mahara et al. (2015) used a novel hetero-bifunctional peptide composed of a collagen-binding region with the integrin a^4^b^1^ ligand, REDV. Peptide-coated small-caliber long-bypass grafts measuring 20–30 cm in length and having a 2-mm inner diameter showed excellent patency in a minipig femoral–femoral crossover bypass model (Mahara et al., 2015). Interestingly, they did not re-endothelialize the graft before implantation. Circulating EPCs might attach on the grafted long-bypass and play a major role in the rapid intima formation in the bypass (Mahara et al., 2015). For rat liver organ engineering, REDV fused elastin-like peptide increased the attachment of ECs, and also spreading, proliferation and peptide conjugation dramatically reduced platelet adhesion (Devalliere et al., 2018). Although the success of coating trials has not yet been proven for microvascular engineering, there is a potential for generating functional bioengineered lung microvessels by preserving patency and preventing leakage (Figure 3).

Many controversial issues remain, which must be confronted for the future success of whole lung engineering. Anastomosis of bronchial circulation and the lymphatic system currently have not been achieved in animal studies. In addition, bronchial circulation has not been achieved in rodents, which limits the ability to study the vascular bed in current models of decellularized rodent lungs (Stabler et al., 2015). It is well known that bronchial arteries supply oxygenated blood to the lung bronchus while the connective tissue of alveolar septa and lymphatic vessels move out extra fluid from connective tissue by negative osmotic pressure, which keeps the alveolar space dry. However, anastomosis of these vessels is technically impossible. Given that the blood flow of the bronchial artery accounts for only 1–2% of total cardiac output in humans (Hall, 2016) and that anastomosis has not been performed in the clinical lung transplantation, lymphatic anastomosis might be unnecessary.

Recellularization using own cells on a decellularized scaffold might lead to clinical transplantation without immunosuppressant. The main targets of the acute or chronic rejections are vascular ECs and the sensitized immunocompetent cells attacking ECs of the vasculature. Thus, if own cells can be used for reendothelialization, theoretically, the immunoreaction will be dramatically reduced and any rejection will be easily controlled. However, the preparation of enough own cells might be difficult because ECs, particularly microvascular ECs, constitute approximately 100 billion cells in a human lung or 500 × 10^6^ cells in a rat lung (Stone et al., 1992; Calle et al., 2014). Because we have not achieved real success in engineered lung transplantation even in rodent models, immunoreaction in the engineered microvascular niche is still unknown.

Another approach to providing lungs for transplantation into humans is to use lung scaffolds from other species. The idea of using organ transplantation across species is problematic because of the differences in anatomic location and size of lungs between species. Further, tissue trimming might be needed when too large xenogeneic lungs are used for transplantation. For example, the porcine right lung has a specific tracheal bronchus with a cranial lobe (Nakakuki, 1994), which would be sacrificed in a right lung transplantation if tissue trimming was necessary. The shape of the lung including vascular routes is also different between the species. Therefore, animal transplantation studies focusing on transplanting pig lungs into primate recipients would be necessary to establish optimal surgical techniques (Tsuchiya et al., 2014b).

Synthetic approaches for bioengineering lung vasculature structures are currently underway. The latest report documents the formation of a 1 cm size vascularized alveolar sac with tidal ventilation (Grigoryan et al., 2019). The final goal of such a model is to make a fully functional respiratory structure. However, the final shape of the model will presumably be similar in structure to the native lung, which has billions of small sacs and surrounding capillaries. In addition, the functional bioengineered lung will ultimately be placed in the thoracic cavity and will need to be anastomosed to bronchial and pulmonary circulation. Otherwise, the engineered lung will not be able to interact with surrounding organs such as the heart, respiratory muscles, and vocal cord. If the future bioengineered lung is made from a scaffold, a decellularized animal scaffold seems at this time to be superior to a synthetic scaffold in regard of natural structure. If an advanced synthetic scaffold does become achievable in the future, the methods of organ engineering using decellularized scaffolds as outlined in this review will be applicable to future synthetic strategies.

In 2018, Cortiella’s group reported an interesting transplantation model within species of bioengineered pig lungs (Nichols et al., 2018). Cultured autologous cells were harvested from a resected own left lung 1 month before transplantation and they were used to recellularize an acellular pig lung scaffold. During 30 days of bioreactor culture, they induced systemic vessel development by adding autologous primary lung cells, primary lung derived vascular cells, primary tracheal–bronchial cells, MSCs with its supernatant, M2 macrophages with its supernatant, and microparticle (MP) delivery of VEGF with hydrogel delivery of platelet-rich plasma (PRP), fibroblast growth factor 2 (FGF2), and keratinocyte growth factor (KGF). After the lung bioengineering, they anastomosed the main bronchus of an engineered lung to the recipient’s main bronchus. However, they did not anastomose the pulmonary artery. As a result, the engineered lung established collateral vascularization as early as 2 weeks after transplant, and partial alveolar formation was observed in the atelectatic lung. There was no indication of transplant rejection. This result indicates that airway function might be achieved by using a mixed population of primary lung cells and several trophic factors. However, the most important point of this study is that they did not anastomose the pulmonary artery of the transplanted bioengineered lung. From the first report of the bioengineered lung transplantation models (Ott et al., 2010; Petersen et al., 2010), anastomosed vessels immediately caused vascular obstruction and organ failure. Therefore, anastomosis of the pulmonary artery might be avoided in this pig bioengineered lung transplantation study. For vascular engineering, full endothelialization with complete intercellular barrier function is required to prevent coagulation activation. Those facts indicate that establishment of functional vascular circulation is the fundamental task to be achieved for successful whole organ engineering.

## Conclusion

Regenerative medicine started from cell-based therapies and today some types of cells are now available in clinical use. The clinical trials of the second generation of tissue-based therapies have also started. Currently, tissue engineering methods have now moved to organoid studies. However, *de novo* whole organ engineering is not realistic because it will take decades in order to generate a usable organ size. In addition, fabrication of whole organs from artificial materials is still in early stages and will be expensive (Grigoryan et al., 2019).

The cell replacement method using an organ scaffold is the current accepted strategy for making organs and it seems to be more realistic for generating usable organs. The method of recellularization of a lung scaffold should take only a week and if complete recellularization can be achieved, would mean the method is easier, faster, and cheaper than other strategies. The most difficult hurdle in bioengineering a lung is in regenerating the microvascular niche with capillary passage and reestablishment of the ABB without leakage. The establishment of the microvascular niche in the engineered organ is the main obstacle that needs to be addressed for a breakthrough in whole organ engineering including lung regeneration.

## Author Contributions

All authors contributed to writing the manuscript.

## Conflict of Interest

The authors declare that the research was conducted in the absence of any commercial or financial relationships that could be construed as a potential conflict of interest.
